# In vivo migration of labeled autologous natural killer cells to liver metastases in patients with colon carcinoma

**DOI:** 10.1186/1479-5876-4-49

**Published:** 2006-11-14

**Authors:** Lina Matera, Alessandra Galetto, Marilena Bello, Cinzia Baiocco, Isabella Chiappino, Giancarlo Castellano, Alessandra Stacchini, Maria A Satolli, Michele Mele, Sergio Sandrucci, Antonio Mussa, Gianni Bisi, Theresa L Whiteside

**Affiliations:** 1 Dept. of Internal Medicine, University of Turin, Italy; 2 University of East Piedmont "Amedeo Avogadro", Novara, Italy; 3 Dept. of Biological and Clinical Science, S. Luigi's Hospital, Orbassano, Italy; 4 S.C.D.U. of Nuclear Medicine 2, Molinette Hospital, Turin, Italy; 5 Medical Oncolocy, COES, Molinette Hospital, Turin, Italy; 6 Flow Cytometry Unit, Laboratory of Pathology, Molinette Hospital, Turin, Italy; 7 Dept of Surgical and Medical Disciplines, University of Turin, Italy; 8 S.C.D.U. of Surgical Oncology, University of Turin, Italy; 9 University of Pittsburgh Cancer Institute, Pittsburgh, PA, USA

## Abstract

**Background:**

Besides being the effectors of native anti-tumor cytotoxicity, NK cells participate in T-lymphocyte responses by promoting the maturation of dendritic cells (DC). Adherent NK (A-NK) cells constitute a subset of IL-2-stimulated NK cells which show increased expression of integrins and the ability to adhere to solid surface and to migrate, infiltrate, and destroy cancer. A critical issue in therapy of metastatic disease is the optimization of NK cell migration to tumor tissues and their persistence therein. This study compares localization to liver metastases of autologous A-NK cells administered via the systemic (intravenous, *i.v*.) versus locoregional (intraarterial, *i.a*.) routes.

**Patients and methods:**

A-NK cells expanded *ex-vivo *with IL-2 and labeled with ^111^In-oxine were injected *i.a*. in the liver of three colon carcinoma patients. After 30 days, each patient had a new preparation of ^111^In-A-NK cells injected *i.v*. Migration of these cells to various organs was evaluated by SPET and their differential localization to normal and neoplastic liver was demonstrated after *i.v*. injection of ^99m^Tc-phytate.

**Results:**

A-NK cells expressed a donor-dependent CD56^+^CD16^+^CD3^- ^(NK) or CD56^+^CD16^+^CD3^+ ^(NKT) phenotype. When injected *i.v*., these cells localized to the lung before being visible in the spleen and liver. By contrast, localization of i.a. injected A-NK cells was virtually confined to the spleen and liver. Binding of A-NK cells to liver neoplastic tissues was observed only after *i.a*. injections.

**Conclusion:**

This unique study design demonstrates that A-NK cells adoptively transferred to the liver via the intraarterial route have preferential access and substantial accumulation to the tumor site.

## Background

Effector functions mediated by cells of the immune system are thought to play a crucial role in the control of tumor development and progression. However, it has been difficult to distinguish the part played by individual immune cell subsets in these processes. T cells that mediate tumor-specific responses are generally considered to be the major anti-tumor effectors. However, cells responsible for mediating innate or natural immunity have been recently recognized to also contribute to anti-tumor defense. Extensive investigation of tumor growth and metastasis in animal models suggests that NK cells are early participants in the immune response and are particularly effective in eliminating blood-borne metastases [1,2]. In contrast, T cells are the effector cells responsible for specific, long-lasting immunity.

In addition to their direct anti-tumor functions [3-7], NK cells can mobilize adaptive immune response [8] presumably by promoting the maturation of dendritic cells (DC) [9-14] and by providing them with antigenic material derived from tumor cell lysis [15,16]. DC mediate both protective and autoimmune responses, depending on their maturational state and soluble factors present in the tissue microenvironment [17,18]. In the liver, for example, Kupffer cells and LSEC constitutively express the anti-inflammatory cytokines, IL-10 and transforming growth factor beta (TGF-β), while hepatocytes secrete IL-10 in response to autocrine and paracrine TGF-β. These cytokines could inhibit maturation of liver DC [19,20]. Therefore, delivery of NK cells to the liver milieu could shift the balance from tolerogenic to immunogenic conditions. The hypothesis we are entertaining is that NK cell-mediated DC maturation in the liver could alter the suppressive local environment, enhance immune responses, and lead to the elimination of tumor cells metastasizing to the liver.

The efficacy of adoptive immunotherapy of solid tumors depends on the localization of therapeutic cells to tissues. This in turn is influenced by the route of administration and access of the cells to tumor. Liver metastases are solely fed by the hepatic artery, and injection of chemotherapeutic agents [21] and immune cells [22] via this route has proven to be effective in controlling the spread of tumors of the digestive tract, whose initial metastasis site is the liver.

The success of adoptive immunotherapy is also critically dependent on the migratory properties of the effector cells and their ability to access tumor cells *in situ*. High expression levels of molecules belonging to the integrin and to the human-leukocyte-function-associated antigen families, namely LFA-1 and very late antigen (VLA)-4, which interact with adhesion molecules on the tumor endothelium, is a characteristic feature of activated NK cells [23]. NK cells have the ability to migrate to, infiltrate and kill cancer cells in solid tumor tissues [24,25] and eliminate established tumors and metastases. Based on their selective ability to rapidly (1–5 h) respond to 22 nM IL-2 by a temporal adherence to solid surface, we have previously defined subpopulations of NK cells, namely activated A-NK cells, which constitute 4–30% of fresh peripheral blood NK cells and are notably different from the non-adherent IL-2 activated cells (NA-NK). A-NK cells produce significant levels of the DC-maturating cytokines IFN-γ, IL1-β and TNF-α and display potent anti-tumor activities *in vivo *and *in vitro *[26].

Injection of A-NK cells into the hepatic artery may be an ideal approach to treating tumors metastasizing to the liver. Although this approach to therapy has been previously used, no formal evidence exists for the superior tumor infiltrating capacity of intraarterially (i.a.)-delivered versus intravenously (i.v.)-delivered A-NK cells. Here we compare in the same patient the efficiency of tumor localization of *ex vivo *generated clinical grade A-NK cells delivered via locoregional i.a. versus i.v. injections.

## Patients, materials and methods

### Protocol design and patient's characteristics

Three patients with colon carcinoma metastasizing to the liver only were enrolled in this study, which was approved by the Italian Ministry of Health and by the Ethical Committee of Turin Molinette's Hospital and the Piedmont Region. Informed consent was obtained from all patients. The patients' characteristics and previous therapy are listed in Table 1. For delivery of A-NK cells, the hepaticarterialcatheter implanted for chemotherapeutical purposes was used. At the time of A-NK-cell injection, all patients had discontinued chemotherapy for at least one month. No disease progression had occurred between the first (i.a.) and the second (i.v.) A-NK administration. Patients had 160 ml blood drawn twice for each of two A-NK cell preparations.

**Table 1:** Patients' characteristics

**Patient**	**Sex/Age**	**Pathology**	**Site of metastasis**	**Previous treatment**
1	M/57	Colon carcinoma	Liver	5FU/FUTR
2	M/62	Colon carcinoma	Liver	5FU/FUTR
3	M/60	Colon carcinoma	Liver	5FU/FUTR

### A-NK cell generation

Cells were generated for therapy under conditions conforming to current good manufacturing practice (GMP), *i.e*., using animal-free serum components and reagents approved for human therapy and accompanied by certificates of analysis (COA). PBMC were isolated from venous blood by Ficoll-Hypaque (Lymphoprep, 1.077 g/ml; Nycomed Pharma, Oslo, Norway) gradient centrifugation and washed in RPMI 1640 (Gibco cat. No. 61870-028) containing 1% (v/v) defibrinated autologous plasma (DAP). The PBMC were suspended at the concentration of 1 × 10^7 ^cells/ml in RPMI 1640 containing 10% DAP, 100 U/ml penicillin, 2 mM L-glutamine, 3 mg/ml sodium bicarbonate, Pen/strep (100 U/ml) (culture medium) and 5 mM phenylalanine methyl ester (PME) at 7.0–7.2 pH. Following a 30 min incubation at RT to deplete monocytes, cells were extensively washed in medium, counted and suspended in the adherence medium supplemented with 1 mM CaCl_2_, 1 mM MgCl_2_, 100 U/ml heparin and 6,000 IU/ml of rIL-2 (Chiron s.r.l., Siena, Italy), obtained from the local Molinette's Hospital Pharmacy and designated for human use. After 3 h at 37°C in a 5% CO_2 _atmosphere non-adherent cells were removed by washing (x3) with pre-warmed medium, and adherent cells (A-NK cell precursors) were counted in four sample flasks with a grid bottom and multiplied for the total number of culture flasks. A feeder layer consisting of rIL-2 (50 IU/ml)-stimulated monocyte-depleted PBMC irradiated at 5,000 rads suspended in culture medium containing 1 × 10^6^/ml of Con A (10 μg/ml) and 6,000 IU/ml rIL-2 was added to each flask. Cells were incubated for 15 days and were fed every four days with rIL-2 (6,000 IU/ml) culture medium to adjust cell density to 1.5 × 10^6^/ml.

### A-NK cell labeling and administration

A-NK cells were harvested, washed, counted and their phenotype was determined. An aliquot was sent for sterility analysis. The remaining cells were suspended in 20 ml clinical grade 0.93% NaCl solution containing 25% DAP, 6,000 IU/ml rIL-2 and 37 MBq ^111^In-oxine (Altana Pharma, Milan, Italy). A-NK cells were generated twice for each patient and the same number of cells was labeled on both occasions. After 15 min at room temperature cells were washed twice in 0.93% NaCl saline containing 25% DAP and 6,000 IU/ml rIL-2. The cell pellet was then suspended in 10 ml 0.93% NaCl containing 20% human albumin (clinical grade) and cells were injected i.a. After 30 days, A-NK cells were again administered, this time i.v. Paracetamol(Acetaminophen) (Perfalgan, UPSA Lab, Braine-l'Alleud, Belgium) (1 g) and 1 × 10^7 ^rIL-2 were given s.c. 30 h before and 48 h after these injections. Each patient was injected with the same number of labeled cells, generated in separate cultures at each of the two time points.

### Evaluation of labeling efficiency

Labeling efficiency was defined as the ratio between free and cell-bound tracer, evaluated in a gamma counter as the cpm of radioactivity contained in the washing supernatant and in the cell pellet.

### A-NK cell migration evaluation

Planar whole-body and single-photon emission-computed tomography (SPET) acquisitions were performed by two-head gamma camera (VARICAM-ESCINT, General Electric, Milwankee, WI, USA) equipped with "medium energy" collimators for ^111^In, with 20% window and energy peaks at 173 and 247 KeV. Planar whole-body acquisitions were performed at 1,6,24,72 and 96 h and SPET acquisitions at 6 and 96 h.

### Localization of A-NK cells to liver metastases

To estimate the extent of the non-pathological liver parenchyma, patients were given an i.v. injection of ^99m^Tc Phytate (FITATEC) (AMERSHAM SORIN, Saluggia, Italy) before A-NK cell administration. Liver images were acquired after 10 min by the gamma camera equipped with "low energy" collimator with 20% window and energy peak at 140 KeV. ^111^In labeled A-NK images were acquired by the gamma camera equipped with "medium energy" collimators, as described above.

### Phenotypic analysis of A-NK cells

A-NK cells were analyzed by flow cytometry on day fifteen. One million cells were incubated for 20 min at 4°C with mouse anti-human simultest CD3FITC/CD16.CD56PE or with γ_1 _FITC/γ_2a _PE isotype controls (Becton Dickinson, San Jose, CA, USA). Flow cytometric analysis was performed using a FACScan flow cytometer CellQuest (v2.1q) software (Becton Dickinson).

## Results

### Growth and phenotypic characteristics of A-NK cells

In two cultures of A-NK cells generated for every patient, very similar numbers of adherent cells were obtained. However, the number of A-NK cells that expanded after fifteen days of culture were strikingly different in the same patient (Table 2). Phenotyping of the final products showed a patient-specific prevalence of NKT CD16^+^CD56^+^CD3^+ ^(patient #1) or of NK CD16^+^CD56^+^CD3^- ^(patients #2 and #3) cells. This phenotype was observed early in culture and persisted throughout the culture period (not shown).

**Table 2:** Biological characteristics of injected A-NK

**Patient**.	**Injection Site**	**Blood ml**	**PBMC^1^**	**Monocyte-depleted^1^**	**Adherent cells^2^**	**Final A-LAK recovery^3^**	**CD16^+^CD56^+^CD3^+ ^%**	**CD16^+^CD56^+^CD3^- ^%**	**Labeled^4 ^cells/injection**	**Labeling efficiency**
1	i.a.	160	1.2 × 10^8^	8 × 10^7^	6 × 10^6^	5 × 10^8^	56	13	5 × 10^8^	58
	i.v.	160	2.8 × 10^8^	1.5 × 10^8^	5 × 10^6^	1.2 × 10^9^	59	14	5 × 10^8^	56
2	i.a.	160	1.2 × 10^8^	8 × 10^7^	3.2 × 10^6^	1.6 × 10^9^	12	69	5 × 10^8^	62
	i.v.	160	2.5 × 10^8^	9 × 10^7^	3.6 × 10^6^	6 × 10^8^	2	85	5 × 10^8^	67
3	i.a.	160	1.3 × 10^8^	5 × 10^7^	1 × 10^6^	6 × 10^8^	10	60	5 × 10^8^	55
	i.v.	160	1.3 × 10^8^	8 × 10^7^	5 × 10^6^	2.3 × 10^9^	12	69	5 × 10^8^	42

^1 ^Peripheral blood mononuclear cells (PBMC) and monocyte-depleted populations were obtained from blood as described in Material and Methods.

^2 ^Cells obtained after incubation of monocyte-depleted cells in presence of the adhrence medium (Materials and Methods).

^3 ^Number of A-NK cells recovered after fifteen days of cultures of adherent cells with rIL-2 and feeder cells as described in Materials and Methods.

^4 ^A-LAK cells recovered on day fifteen were ajusted at the indicated number and labeled with ^111^In-oxine before being injected i.a. or i.v.

### NA-NK cell labeling efficiency and stability

The mean efficiency of labeling was 56% (range 42–67%) (Table 2).

### Migration of A-NK cells to the liver

**Figure 1:**
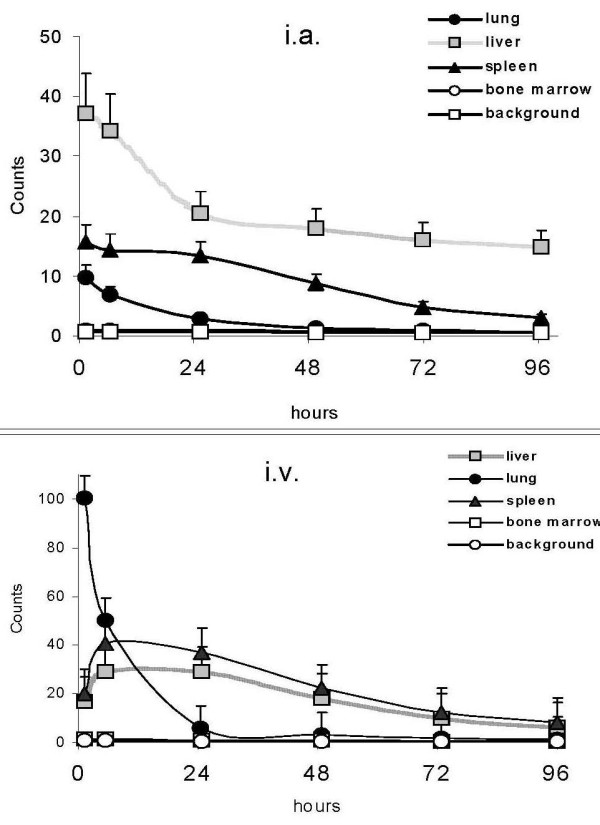
***In vivo migration of A-NK cells***. The same number of ^111^In-labeled A-NK cells were injected i.a. (A) and, after one month, i.v. (B). Organ distribution of radioactivity was monitored at the indicated times (X axis) by planar whole-body acquisition (Y axis). An earlier and greater migration of labeled cells was observed in the liver after i.a. administration. Counts are corrected for isotope decay and refer to the mean ± SE of the three patients.

**Figure 2:**
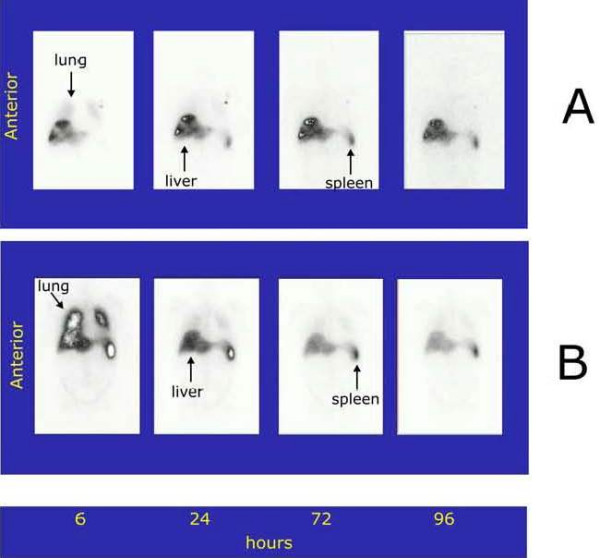
***Representative planar whole-body images of organ migration of ^111^In-labeled A-NK after i.a. (A) and i.v. (B) injections (patient #2)***. ^111^In uptake was observed in the liver soon after the i.a. administration and persisted for up to 96 h.

**Figure 3:**
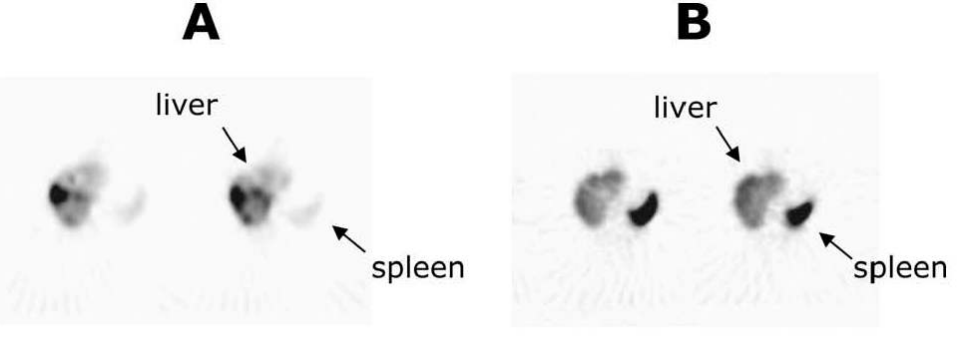
***SPET images from patient #1, 6 h after i.a. (A) or i.v. (B) injection of A-NK cells***. Greater migratory activity of A-NK cells is observed in the liver after i.a. administration. Two consecutive axis sections are shown.

Migration of the i.a. injected A-NK cells and of i.v. injected A-NK cells (after 30 days) were assessed by planar whole-body scintigraphy and SPET. Fig. 1 shows the kinetics of tracer accumulation after i.a. (A) and i.v. (B) injection in the three patients. Very low tracer activity was observed in the lung after i.a. injection, whereas liver labeling was immediate (four-fold and two-fold the lung and spleen labeling respectively) and was still evident at 96 h. Immediate labeling of lung observed after i.v. injection became evident at 24 h and declined thereafter. The representative radioimaging of Fig. 2 illustrates differential progression of ^111^In-labeled A-NK cells from lung to spleen to liver during the observation time after i.a. (A) and i.v. (B) delivery (patient #2). In a 24 h axial section from this patient, SPET showed preferential accumulation of radioactivity in liver compared to spleen six hours after i.a. (Fig. 3A), but not after i.v. (Fig. 3B).

### Localization of A-NK cells to tumor nodules

**Figure 4:**
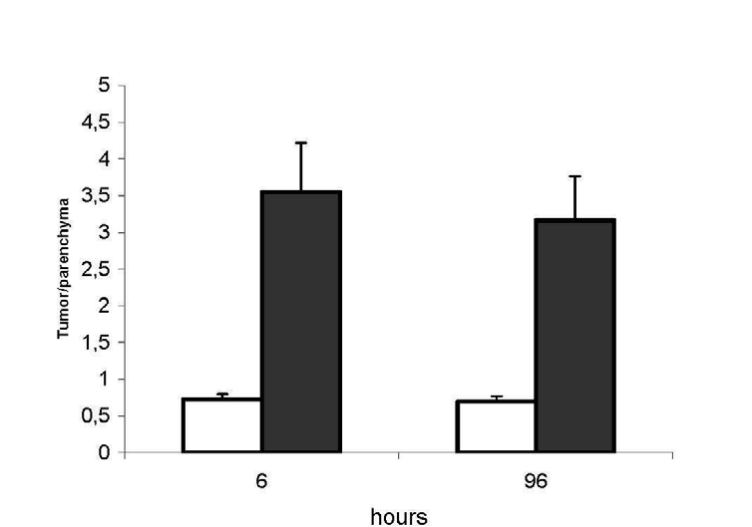
***Normal parenchyma and tumor lesions***. Patients were given ^99m^Tc phytate before i.v. (open bars) or i.a. (full bars) ^111^In-Oxine labeled A-NK administration. The tumor/parenchyma labeling ratio (Y axis) was calculated as the ratio between the ^111^In counts in metastatic (no ^99m^Tc phytate uptake) and normal (^99m^Tc phytate uptake) SPET areas.

**Figure 5:**
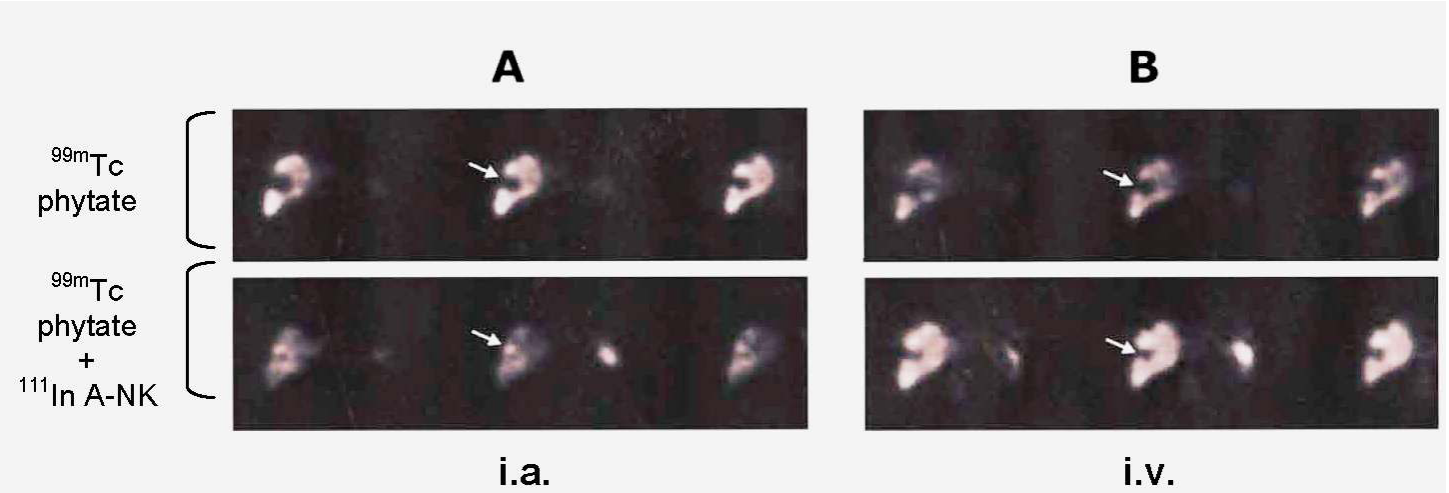
***Tumor migration of A-NK cells***. SPET analysis from patient #1 performed 10 min after ^99m^Tc phytate (top) and 6 h after subsequent i.a. (A, bottom) or i.v. (B, bottom) injection of ^111^In-Oxine-labeled A-NK cells. A "cold" (no uptake) area corresponding to tumor tissue is observed before i.a. or i.v. A-NK injections (A and B, top). This area is ^111^In-positive after i.a. (A, bottom), but not after i.v. (B, bottom) injection of A-NK cells. Three consecutive axial sections are shown.

^99m^Tc phytate is transformed by chelation with serum calcium in vivo into a microcolloid which is taken up by cells of the reticulo-endothelial system, particularly Kupffer cells. ^99m^Tc phytate was given i.v. before A-NK cell delivery to estimate the extent of normal liver parenchyma. ^111^In radioactivity was then evaluated in ^99m^Tc phytate-positive and in ^99m^Tc phytate-negative areas to assess the ratio between tumor-involved and normal tissues. This ratio was >3 for i.a., but remained virtually unchanged at 0.7 for i.v. (Fig. 4, mean ± SE of the values referred to the three patients). A representative SPET image analysis performed in patient #1 (Fig. 5) illustrates ^99m^Tc phytate uptake (top) and ^111^In radioactivity (bottom) after i.a. (Fig. 5A) or i.v. (Fig. 5B) cell delivery. The ^99m^Tc phytate-negative (cold) areas identified by the arrows in Figs. 5A and 5B (top) are positive for ^111^In in Fig. 5A (bottom), but not in Fig. 5B (bottom). The image is consistent with the localization of ^111^In-labeled A-NK cells in the neoplastic hepatic nodule after i.a., but not after i.v. administration.

## Discussion

As the first metastatic site of gastric and colon cancer, the liver is a critical check point for systemic disease progression. The liver is rich in immune cells, including DC precursors, NK cells and T lymphocytes. Generation of CD8^+ ^tumor-specific T cells, which mediate anti-tumor responses in the liver, is enhanced by activated NK cells, which facilitate maturation of DC [8-14]. However, a suppressive liver environment hampers functional maturation of DC and the consequent activation of the adaptive immune response. Therapy with activated NK cells can therefore be envisaged as a means to breaking liver tolerance and to boosting antigen-specific anti-tumor responses. The efficiency of adoptive cellular immunotherapy of cancer strongly depends on the type, number and the route of delivery of effector cells that are expected to reach malignant tissues after their transfer.

We demonstrate that *ex vivo *expanded A-NK cells efficiently home to liver metastases and only accumulate therein, when they gain direct access to the portal system. This route may favor the localization and concentration of immune effector cells in the tumor, since liver metastases are fed with arterial blood. However, preferential localization in the tumor is also likely to depend on the intrinsic homing characteristics of the injected cells. NK cells have a regulated expression of a number of integrins that may play a role in their localization. *In situ *immunohistochemical staining has shown NK-cell localization to the red pulp of the spleen and the sinusoid regions of the liver. Few NK cells are present in other solid organs and, surprisingly, there are relatively few NK cells in lymph nodes [27]. NK cells present in the liver sinusoids are strategically located to detect and kill arriving metastatic cancer cells, and their accumulation at this site is favored by chemokine ligands present in the LSEC [28]. The LSEC cytokine environment, with IL-2 produced by resident T cells [29] and IL-15 and IL-12 produced by Kupffer cells [30], might be expected to potentiate homing and cytotoxic functions of NK cells. Indeed, genes involved in NK-cell homing, cytotoxicity and cytokine secretory functions are overexpressed in hepatic NK cells compared to blood NK cells [28], but their expression profile is similar to that of IL-2-activated blood NK cells. It is, therefore, very likely that the radioactivity detected in the metastatic areas of the liver is due to interactions between labeled A-NK cells and the tumor LSEC.

The ability of A-NK cells to penetrate the tumor tissue is relevant to their potential use as carriers of anti-tumor drugs and as direct killers of tumor cells. In experimental *in vivo *tumor models, NK cells are more frequently found to localize tumors which are also sensitive to NK cell killing *in vitro *[27]. Besides direct anti-tumor activities of NK cells, their localization in the liver tumor area would be important for the maturation of DCs, possibly through the production of IFN-γ and other cytokines.

Two A-NK subsets predominated in our three patients after a fifteen-day expansion: CD16^+^CD56^+^CD3^+ ^in patient #1 and CD16+CD56+CD3- in patients #2 and #3. Adhesion to tissue cells expressing the relevant receptors is the main property of these cells, and it is conferred, among other molecules, by N-CAM, which is present on NK cells and a subpopulation of NKT cells [31,32]. NKT cells are the population best represented in the liver [33]. In our study, preferential ^111^In labeling of these A-NK subpopulations (both NK and NKT cells) in the metastatic compared to normal liver was observed.

We found a prompt localization of A-NK cells to the lung after i.v. injection, as also shown by other *in vivo *studies [24,25,35]. Transit of NK cells to the lung via the peripheral blood circulation is likely to play a surveillance role *in vivo*, since radiolabeled tumor cells injected i.v. are eradicated by NK cells before they enter the lung [27]. Radioactivity detected in our study is most probably attributable to cell-bound rather than to cell-free tracer for two reasons. First, the absence of excretion of radioactivity over the observation period (ref [25] and our data not shown) indicates extended survival of injected A-NK cells. Second, free tracer would not have been taken up by the tumor and was not taken up after i.v. administration (Fig. 5B bottom). The half-life of adoptively transferred NK cells is about 7–10 days [36]. This is particularly true in the permissive environment of liver sinusoids.

## Conclusion

We have performed an imaging study of the biodistribution of *ex vivo *activated, adoptively-transferred autologous A-NK cells and demonstrated their differential localization in normal hepatic parenchyma and liver metastases. The unique opportunity offered by this study of comparing different administration routes in the same patient, allowed unambiguous demonstration that A-NK cells localize to liver metastases only when injected locoregionally. This finding has important implications for the design of future immunotherapy protocols based on the principle that autologous A-NK cells adoptively transferred to the liver via the arterial route have preferential access and substantial accumulation to the tumor site.

## Abbreviations

A-NK: adherent Natural Killer; i.v.: intravenously; i.a.: intraarterially; LSEC: liver sinusoidal endothelial cells; PME: phenylalanine methyil ester; cpm: counts per minute; SPET: single-photon emission-computed tomography.

## Authors' contributions

LM had the idea for, coordinated and analysed experimental data, obtained funding and wrote the report.

AG contributed to protocol design, was in charge with the experiments and helped to draft the manuscript

SS and AM contributed to the protocol design.

IC and MAS contributed to protocol design, patients enrolment, follow up and clinical care.

GB, MB, CB and GC were in charge for labelling and infusion of A-NK cells and imaging study.

AS performed phenotypical analysis of ex-vivo expanded A-NK cells.

MM contributed in the graphic elaboration of A-NK migration images.

TLW designed the A-NK protocols, critically analysed the results and revised the paper.

All authors read and approved the final manuscript.
